# Daily Life Studies on Dynamic Within-person Fluctuations of Self-efficacy in the Physical Activity Context: A Scoping Review

**DOI:** 10.1186/s40798-025-00973-z

**Published:** 2026-02-07

**Authors:** Anna Vogelsang, Claudio R. Nigg, Ulrich W. Ebner-Priemer, David Haag, Markus Reichert

**Affiliations:** 1 Department of eHealth and Sports Analytics, Faculty of Sport Science, Ruhr- University Bochum (RUB), Gesundheitscampus Nord 10, 44801 Bochum, North Rhine-Westphalia Germany; 2 Department of Health Science, Institute of Sport Science, University of Bern, Bern, Switzerland; 3 Department of Psychiatry and Psychotherapy,, Central Institute of Mental Health, Medical Faculty Mannheim/Heidelberg University, Mannheim, Baden- Wuerttemberg Germany; 4 Ludwig Boltzmann Institute for Digital Health and Prevention, Salzburg, Austria; 5 Department of Psychology, Paris-Lodron-University of Salzburg, Salzburg, Austria; 6 Center for Cognitive Neuroscience, Paris-Lodron-University of Salzburg, Salzburg, Austria; 7 Digital Health Information Systems, Center for Health & Bioresources, AIT Austrian Institute of Technology GmbH, Graz, Austria; 8 Department of Sport and Exercise Science, Faculty of Natural and Life Sciences, University of Salzburg, Salzburg, Austria

**Keywords:** Health behavior theory, Dual process models, Intraclass-correlation coefficient, Intensive longitudinal studies, Social cognitive theory, Ecological momentary assessment, Ambulatory assessment

## Abstract

**Background:**

Cutting-edge dual process health behavior theories propose micro-temporal within-person processes to be critical drivers of physical activity participation. Self-efficacy is the pivotal motivation-oriented correlate of physical activity, a key component across the most prominent health behavior change theories, and has predominantly been researched as stable interpersonal ‘trait’ factor. However, the micro-temporal within-person ‘state’ perspective on self-efficacy remains uncharted.

**Objectives:**

To tackle this research gap, we conducted a scoping review and examined (1) time-sensitive (i.e., assessment time span) and (2) theory-conform operationalization of self-efficacy measures as well as (3) within-person variance reports from ecological momentary assessment studies in the physical activity context among healthy adults.

**Methods:**

A scoping review of English articles using PsycINFO, PsycArticles, PSYNDEX, SPORTDiscus and PubMed was conducted up to September 2025. Eligible studies focused on (1) physical activity in (2) healthy adults aged + 18 years and (3) applied multiple within-day, daily or weekly assessments of self-efficacy. Findings were summarized through quantitative analysis of the evidence.

**Results:**

A total of 13 studies was included. Most studies assessed self-efficacy through multiple assessments per day and with a focus on the near future (i.e., next few hours post ecological momentary assessment). The 13 identified self-efficacy items were operationalized according to self-efficacy theory, but varied in semantics, psychometrics, and source. Five studies reported intraclass correlation coefficients that revealed self-efficacy within-person variance to range between 51% and 89%.

**Conclusions:**

Given the pivotal role of self-efficacy across various health-behavior theories and the recent relevance attributed to micro-temporal within-subject processes, thus far surprisingly few studies researched how self-efficacy unfolds within-persons across time. However, the few studies identified provide initial evidence that self-efficacy varies within individuals across time in everyday life, including a tendency towards higher within-person variance for momentary versus day level assessments, and thereby empirically supporting dual process models. Items were assessed dynamically using repeated measures per day and according to theory but differed in conceptual and semantic features. Future research is encouraged to further investigate how self-efficacy unfolds across time, by testing various sampling strategies and applying advanced designs to shed light on the precise timing of effects and to inform adaptive and expedient intervention development.

**Supplementary Information:**

The online version contains supplementary material available at 10.1186/s40798-025-00973-z.

## Key Points

- We reviewed the latest ecological momentary assessment (EMA) studies examining self-efficacy fluctuations for physical activity, and we provide in-depth insights towards time-dynamical research in the health behavior field.
- EMA items reviewed were operationalized according to self-efficacy theory, but they differed in semantic and conceptual characteristics due to necessary adaptations for capturing context specific self-efficacy (i.e., related to assessment time span and self-efficacy type) rather than reflecting measurement inconsistencies.
- Most EMA studies employed multiple electronic diary assessments per day aiming to capture self-efficacy across a few hours post assessment. Future research should combine various sampling strategies to obtain a more comprehensive, dynamic picture of how self-efficacy unfolds in daily life to shed light on the precise timing of effects.
- Overall, self-efficacy appears to fluctuate within rather than across days. These finding call for future studies to rationalize sampling scheme decisions, in order to optimally capture fluctuations in self-efficacy within a day for expedient and individualized health behavior change.

**Supplementary Information:**

The online version contains supplementary material available at 10.1186/s40798-025-00973-z.

## Introduction

Globally approximately one in three adults suffers from multiple health conditions such as coronary heart disease, cancer, and major depressive disorder [1, 2]. The risk of developing such a condition can be substantially minimized by health-protective behaviors, especially by physical activity [3]. Although recommended levels of physical activity can be attained through both structured exercise and accumulated via relatively brief physical activity bouts across the day, the worldwide activity participation level is critically insufficient [4]. To counteract this inactivity pandemic, motivation-oriented constructs are awarded a particularly important role as driver of increased physical activity levels [5].

Self-efficacy, an individual’s belief in their ability to engage in and arrange the required actions to attain desired outcomes [6], presents itself as a key motivation-oriented construct, repeatedly being evidenced to predict physical activity [7]. Furthermore, recent research highlights the role of self-efficacy as a mediator through which physical activity minimizes psychological disorders [8]. Self-efficacy is embedded in most of the existing health behavior theories and is particularly prominent within the well-recognized social-cognitive theory (SCT [9]). SCT builds upon the concept of reciprocal determinism (also called triadic reciprocity) in which behavior, environment, and personal factors interact and dynamically shape one another [6].

Traditional models of health behavior change, including SCT, and the theory of planned behavior [10] predominantly concentrate on static individual-level constructs seeking to comprehend how interindividual (i.e., between-person) differences in psycho-social factors account for between-person differences in overall physical activity levels [11, 12]. For example, reviews and meta-analyses show that participants reporting higher self-efficacy scores engage in more physical activity compared to those with lower self-efficacy scores [13–15]. While such between-person approaches have dominated behavioral research for years, they are now regarded to as being flawed in part and conceptually problematic [16]. Moreover, cutting-edge dual process health behavior theories (e.g. the WANT model -Wants and Aversions for Neuromuscular Tasks [17], the dual-mode theory [18] or the affective-reflective theory [19]), propose that behavior is steered by reflective, conscious decision making processes and automatic, unconscious influences on action [20–22], and increasingly emphasize micro-temporal within-person processes to be critical drivers of physical activity participation [19].

In practice, the associations between a person’s average self-efficacy and his/her average physical activity level across time may indeed not reflect that person’s momentary levels of self-efficacy and physical activity. Put simply, higher self-efficacy scores on Monday at 7 pm may encourage a person to exercise, compared to lower self-efficacy scores on Monday 1 pm making him/her skip the exercise class [5]. As such, between-person data may be limited in covering self-efficacy fluctuations throughout the day (i.e., across minutes, hours) that collectively contribute to accumulating physical activity levels [23, 24].

While SCT is classified as a conceptual model that proposes several dynamic feedback loops between a person’s reflections, environment, and behavior, it qualifies well for a dynamical model, a potential that has often not yet fully been utilized [12]. Therefore, research on time-varying circumstantial determinants, and on how individuals’ reactions to them are connected to physical activity engagement presents an essential next step to arrive at expedient and adaptive lifestyle physical activity programs [25].

To shed light on these dynamic processes, intensive longitudinal data present a promising approach. Secondary to rapid technological advances, mobile or social sensing as well as ecological momentary assessment (EMA [26]), thanks allow for collecting frequently repeated within-person assessments with a high timely density (hourly or daily) in real-life settings [27]. Examining the resulting fluctuations of social-cognitive predictors within-person will allow us to find answers to questions of when associations occur, how long they stay and when they subside [28, 29]. Empirical evidence on the momentary within-person fluctuations of variables and their influence on physical activity derived from intensive longitudinal data exists mostly for momentary affective states that are related to physical activity [30]. Interestingly, previous research already manifested that social-cognitive determinants fluctuate within individuals [31, 32], and a recently emerging systematic review, investigating day-level and within-day associations between physical activity and sedentary behavior, documented that daily self-efficacy and intentions are crucial factors steering physical activity and sedentary behavior in everyday life [33].

However, there is no in-depth review focusing on self-efficacy as a putative micro-temporal driver of physical behavior, e.g., investigating assessment-related aspects of self-efficacy as a within-person predictor, such as the items’ time scales (e.g., day vs. within-day level), the sampling scheme and whether more assessments per day reveal more within-person fluctuations as opposed to just one assessment. These insights are crucial to determine whether day-level associations for self-efficacy (and intention as found in [33]) and physical activity also hold true at the micro-temporal level. Furthermore, they are needed to develop validated EMA assessment tools for self-efficacy and other social-cognitive determinants of physical activity behavior. To this end, this scoping review intends to examine (1) time-sensitive (i.e., assessment time span) and (2) theory-conform operationalizations of self-efficacy measures as well as (3) within-person variance reports from EMA-based studies in the physical activity context among healthy adults. The overall goal is thus to synthesize the currently available evidence of self-efficacy variations in the physical activity context. In particular, the assessment time span, covering momentary, daily and weekly assessments of self-efficacy items, the theory-conform operationalization of self-efficacy focusing on word choices and word meaning in items (i.e., lexical semantics), and the statistical reporting (i.e., within-person variance reports) of EMA-based studies were researched. Weekly assessments were added to also account for self-efficacy fluctuations of those who exercise regularly (i.e., weekly) but not daily. Given that prevailing evidence pertaining to within-person fluctuations in self-efficacy is still in its infancy, a scoping review appeared to provide the needed systematic and iterative format to detect and synthesize the existing evidence and to fill this relevant gap in the literature.

## Methods

This review was guided by the PRISMA scoping review extension checklist [34]. The checklist, specifying how and where the review complies with the guidelines, is attached as supplementary data. No protocol was registered a priori.

### Eligibility Criteria

Studies were eligible for inclusion if they met the following criteria: focused on physical activity and exercise behavior in healthy adults (i.e., non-clinical populations) aged 18 + years; incorporated multiple within-day, daily, or weekly assessments of self-efficacy (i.e., used an EMA methodology of any length and contained a measure of self-efficacy); and were published in English. Studies including an intervention were included as well as studies involving participants with a diagnosed physical or mental health condition who were not recruited into the study based on their condition (e.g., clinical level of depression but where this condition was not an inclusion criterion). Furthermore, next to micro-temporal assessments (i.e., momentary and daily), weekly assessments have been included to account for self-efficacy fluctuations of those who exercise regularly (e.g., weekly) but not daily. No restriction was set on publication date or geographical location. Laboratory studies were excluded from the review as well as studies including clinical populations (that were recruited based on their indication), not being peer-reviewed, or not published in English or where no full text could be obtained. Studies assessing perceived behavioral control were excluded, given that this entails the subcomponents self-efficacy and controllability, which results in differences in how perceived behavioral control is operationalized [35]. Since the review’s focus is on self-efficacy, we decided to exclude perceived behavioral control to arrive at a clearer vision of how self-efficacy is assessed. Additional guidance on inclusion criteria is provided in the Electronic Supplementary Material (ESM Sect. 1.1).

### Information Sources and Search Strategy

Searches were conducted using PsycINFO, PsycArticles, PSYNDEX, SPORTDiscus and PubMed. The initial search was carried out in August 2023, yielding eight articles, and repeated using the same criteria in January 2024 and September 2025, resulting in an additional two (i.e., 2024) and three (i.e., 2025) publications, for a total of 13 articles. Ten of those articles used individual datasets, while three studies used data from the same project: [5, 36–40]. The search strategies were drafted by AV and further refined through team discussion. The electronic database search was complemented by searching reference lists of retrieved articles, and previously published review articles reporting on social-cognitive determinants in the exercise and physical activity context using EMA (e.g [33]). Likewise, manual searches were undertaken in databases, journals, and publication lists for authors who already published in this area, to ensure completeness of records. Furthermore, four authors were contacted privately, as articles were not available online or self-efficacy items were not published in the articles. Except for one, all authors replied and provided the requested full texts or items, respectively. Duplicates were either removed directly by the database or manually by AV. The final search results were exported into Microsoft Excel.

### Search

The final search strategy per database can be found in the ESM (Sect. 1.2). Terms were searched in titles and abstracts as free-text terms or as index terms, as appropriate. Three groups of terms were combined: the first group of terms was relevant to EMA and within-person study designs; the second group referred to physical activity and exercise behavior and the last group was relevant to self-efficacy. The following search terms were used: (1) (“ecological momentary assessment*”) OR (“intensive longitudinal”) OR (“ambulatory assessment*”) OR (“experience sampl*”) OR (“daily diar*”) OR (“ecological momentary intervention”) OR within-person OR within-subject* OR idiographic OR intraindividual OR “real time data capture” OR “daily life research”; (2) “physical activit*” OR exercis*; (3) self-efficacy. Truncations were applied to include multiple word endings (e.g., diar* would capture diary, diaries etc.).

### Selection of Sources of Evidence

**Fig. 1:**
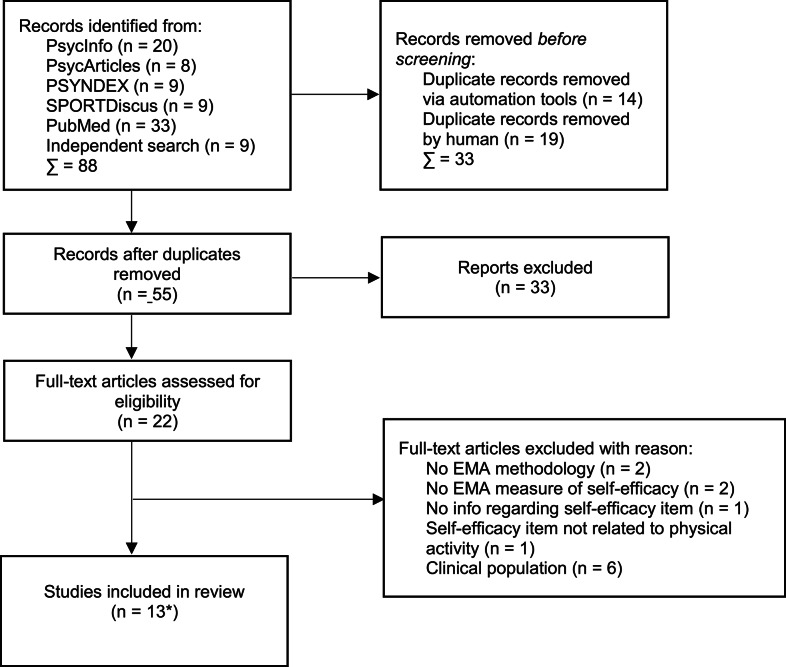
Flow chart of reviewed studies according to PRISMA [34]. * An update of the literature search revealed another three articles, resulting in 13 studies to be included

Titles and abstracts were evaluated for eligibility in accordance with the screening guidelines in the ESM (Sect. 1.1) by AV. Irrelevant papers were excluded. Papers in which uncertainties about inclusion arose were screened in full. Remaining uncertainties on study inclusion were discussed by AV and MR, referring to the inclusion and exclusion criteria to reach consensus. The final total number of papers in the review was 13 and these were agreed to and read in full (see Fig. 1).

### Data Charting Process and Data Items

A data-charting form using Microsoft Excel was developed by agreement between AV and MR to identify which variables to extract. AV independently charted the data and continuously updated the data-charting form in an iterative process. Data extraction included general study characteristics (ESM Sect. 2.1), time span-related matters of items assessing momentary self-efficacy (Table 1) and statistical reporting (i.e., multilevel variance components; Table 2).

### Synthesis of Results

Results were presented in tabular and descriptive format. At first, studies were scanned to identify study and sample characteristics (ESM Sect. 2.1). The subsequent synthesis of results aligns with the research aims: first, studies were grouped and scanned by the assessment time span they were reporting (Table 1). To classify assessment time spans, the following intervals were defined: the momentary time span, referring to the here and now (e.g., in this moment), the near future time span referring to the next hours post the EMA assessment (e.g., in the next few hours, today), the prospective time span referring to the following day/days/week post the EMA assessment (e.g., tomorrow, in the next days) and the retrospective time span referring to the time prior to the EMA assessment (e.g., since the last beep). Besides these assessment time spans, studies were also scanned regarding their sampling scheme. Second, and regarding the second aim, studies were scanned and grouped according to the operationalization used to assess momentary self-efficacy (Table 1). This will be approached via lexical semantics, which is the study of word meaning and analyzes how single words transfer meaning, how these meanings connect to each other (e.g., synonyms) and how this adds to the meaning of a sentence (i.e., the items). Focusing on lexical semantics is essential to ensure that self-efficacy measures accurately assess the intended constructs, that items are clearly worded, theoretically aligned and free from unintended ambiguities, thereby enabling valid and comparable research findings [41]. In this regard, it is also important to emphasize that potential semantic differences in items will explicitly be evaluated in terms of word choices and meaning, and not in relation to assessment time spans or specific self-efficacy types (as this may result in semantic differences that naturally occur from item adaptation to meet assessment windows or conceptual characteristics). Attention was also paid to the response format, the source of the items (i.e., whether ‘precedents’ exist, i.e., items that have been previously used in studies vs. have been developed for the study) and psychometric information. Moreover, whether additional social-cognitive constructs had been assessed was also checked. Lastly, studies were scanned regarding the statistical reporting of multilevel variance components (Table 2).

## Results

### Selection of Sources of Evidence

Through the primary and secondary search, 121 publications were identified (Fig. 1). Duplicates were either removed manually or via automation tools offered by the databases. A total of 55 articles were subsequently screened (by title and abstract) resulting in 22 publications to be assessed for eligibility. For different reasons, 12 publications did not meet the inclusion criteria (e.g., no EMA methodology applied, self-efficacy not assessed through an EMA measure, information pertaining to the self-efficacy items was missing, self-efficacy was not related to physical activity behavior, clinical population, see Fig. 1). An update of the literature search yielded another three articles. In total, 13 publications were included in the present review, including ten original articles (and datasets) and three articles that used datasets from the same project.

### Characteristics of Sources of Evidence

The general and design-related characteristics of the studies included in this review are provided in the ESM (Sect. 2.1). Table 1 provides an overview of the assessment time span and operationalization related information (e.g., the exact item wording, number of items, response range) pertaining to items assessing momentary self-efficacy. An overview of the statistical reporting of multilevel variance components of self-efficacy measures is presented in Table 2.

### Results of Individual Sources of Evidence

#### Study and Sample Characteristics

A comprehensive overview of the study and sample characteristics is provided in the ESM (Sect. 2.1). It is important to note that, studies that were based on the same dataset were counted only once (i.e., not double), but, are referenced individually. Most studies (*n* = 4) included middle-aged to older adults [12, 42, 43], followed by three studies focusing on adults [5, 36, 37, 40, 44]. Samples tended to be mixed sex, with one study including female participants only [44]. Various theoretical frameworks were applied, with most of them being based on SCT [5, 9, 40, 44, 45] followed by the Health Action Process Approach [36, 37, 46, 47]. All but two study were theory driven [38, 42] and two studies reported including an intervention to foster physical activity behavior [44, 45]. Although the technology used to assess self-efficacy differed, most studies (*n* = 8) used mobile phones for the data acquisition [5, 12, 36, 37, 40, 43–45]. The study length ranged from 4 days [5, 38–40] to 28 days [47], with one study covering 12 weeks [44]. Most studies (*n* = 8) applied accelerometers to assess physical activity behavior [5, 38–40, 43–45, 47].

#### Assessment time Span of Self-efficacy Items

Of the 13 identified self-efficacy items deriving from 10 studies, most items (*n* = 7) focused on the near future time span referring to the next two or few hours [5, 12, 38–40, 42, 43, 45], followed by the prospective time span referring to ‘tomorrow’ [47, 48] or ‘this week’ [44] (Table 1). One study had no time span in its respective items [36, 37] 5. The retrospective time span was not referred to in any study. Regarding the sampling scheme, most studies (*n* = 4) used fixed-interval or signal-contingent with equidistant prompts four [36, 38, 39, 42], or six [12, 43] times a day [5, 40]. Two studies used six [43] to eight prompts per day at random times [5, 40]. Three studies used end-of-day diaries [45, 47, 48], and one study used end-of-week assessments [44].

**Table 1:** **Assessment time perspective and operationalization of self-efficacy items**

Study	EMA duration	Sampling scheme	Operationalization	Assessment time span of items	Response format	Item source	Psychometrics	Additional social-cognitive constructs assessed
Momentary assessments								
Dunton et al. 2009 [42]	14 days	Fixed-interval measurement four times/day (7:45am, 11:45am, 3.45pm, 7:45pm)	How confident are you that you can engage in physical activity that increases your heart rate for at least 10 min during the next few hours?	In the next few hours	10-point response scale (not at all confident – completely confident)	Modified from past research using EMA	–	Mood
Haag et al. 2023 [36]	21 days	Signal-contingent sampling with fixed times (9am, 1pm, 5pm, 9pm) and event-contingent sampling	How strongly do you believe you can enact your plan under the given circumstances?	–	Visual-analogue scale (0 = not at all – 100 = very much)	Items were designed for this study		Intentions Planning specificity Intrinsic motivation
Kumar et al. 2025 [37]	21 days	Signal-contingent sampling with fixed times (9am, 1pm, 5pm, 9pm) and event-contingent sampling	How strongly do you believe you can enact your plan under the given circumstances?	–	Visual-analogue scale (0 = not at all – 100 = very much)	Items were designed for this study		Intentions Planning specificity Intrinsic motivation
Maes et al. 2022 [12]	7 days	Signal-contingent, six times per day between 9am and 10pm	In the next two hours, I can move for at least 10 min.	In the next two hours	7-point Likert scale (1 = strongly disagree – 7 = strongly agree)			Intention Physical complaints emotions
Maher et al. 2016 [5]	3 × 4-day measurement bursts with 6 months between bursts	8 prompts per day at random times (6:30am – 10:00pm)	Can you do 10 + min of physical activity sometime within the next few hours even if you start to feel tired?	Within the next few hours	1 = I know I cannot – 5 = I know I can	Based on [30]	Composite score by averaging responses Ω = 0.84	Intention Outcome expectations
			Can you do 10 + min of physical activity sometime within the next few hours even if you get busy?	Within the next few hours				
Maher et al. 2020 [43]	10 days	6 prompts per day at random times between 8am and 8pm	Over the next 2 h, I believe I can stand or move for at least 30 min.	Within the next 2 h	5-point response scale (1 = strongly disagree; 5 = strongly agree)	Adapted from [30, 53]		Intentions
Maher et al. 2025 [38]	3 × 4-day measurement bursts with 6 months between bursts	Signal-contingent, 10 prompts per day tailored to sleep and wake times (i.e., 07:00AM-09:00PM, 08:00AM-10:00PM or 09:00AM-11:00PM)	Over the next hour, I feel confident that I can engage in at least 10 min of physical activity.	Over the next hour	Visual-analogue scale (0 = not at all confident − 100 = very confident)	Adapted from [5, 36]		Intentions Planning
Maher et al. 2025 [39]	3 × 4-day measurement bursts with 6 months between bursts	Signal-contingent, 10 prompts per day tailored to sleep and wake times (i.e., 07:00AM-09:00PM, 08:00AM-10:00PM or 09:00AM-11:00PM)	Over the next hour, I feel confident that I can engage in at least 10 min of physical activity.	Over the next hour	Visual-analogue scale (0 = not at all confident − 100 = very confident)	Adapted from [5, 36]		Intentions Planning
Pickering et al. 2016 [40]	4 days (2 weekdays, 2 weekend days)	Randomly within 8 specific time intervals during the day: 6:30am, 8-10am, 10-12am, 12-2pm, 4-6pm, 6-8pm, 8-10pm	Can you do 10 + min of physical activity sometime within the next few hours even if you start to feel tired?	Within the next few hours	5-point response scale (1 = I know I cannot, 5 = I know I can)	Based on [59]	Composite score by averaging responses (α = 0.91)	Intention Outcome expectations
			Can you do 10 + min of physical activity sometime within the next few hours even if you get busy?	Within the next few hours				
Daily assessments								
Berli et al. 2018 [47]	28 days	End-of-day diary	I am confident that I can be physically active tomorrow even if it is difficult. (daily self-efficacy)	Tomorrow	1 (today not at all true) – 6 (today completely true)	Adapted from scales by [60]		Daily action control Daily intentions Daily action planning
Conroy et al. 2013 [48]	14 days	End-of-day web-based questionnaire (7pm – 4am)	I believe I can accumulate at least 30 min of moderate aerobic activity tomorrow	Tomorrow	1 (not at all confident) – 5 (completely confident)	–	Average of both items (α = 0.90)	Daily PA intentions Attitudes Subjective norms Daily constraints on PA motivation
			I believe I can accumulate at least 15 min of vigorous aerobic activity tomorrow (daily PA self-efficacy belief)	Tomorrow				
Schwaninger et al. 2021 [45]	14 days	End-of-day dairy	I am confident that I can be physically active tomorrow even if it is difficult.	Tomorrow	6-point response scale (1 = today not at all true; 6 = today completely true)	Adapted from [60]		Daily support receipt
Weekly assessments								
Oh et al. 2023 [44]	12 weeks	7 and 14 days post randomization between 11am and 3pm	How much confidence do you have that you will achieve your weekly goal this week?	This week	0 = not confident, 1 = a little confident, 2 = confident, 3 = very confident			

*EMA* ecological momentary assessment

#### Operationalization of Self-efficacy Items

All 13 self-efficacy items were phrased in either terms of ‘confidence to’, ‘can’ or ‘could’ (Table 1). Five out of 13 items were phrased as questions (e.g., ‘How confident are you that you can engage in physical activity that increases your heart rate for at least 10 min during the next few hours’ [42]), whereas seven items were phrased as statements (e.g., ‘I am confident that I can be physically active tomorrow even if it is difficult’ [47]), . Furthermore, four items entailed a conditional sentence (e.g., I am confident that I can be physically active tomorrow even if it is difficult; [5, 40, 45, 47]), with conditions varying between ‘even if it is difficult’ [45, 47], ‘even if you start to feel tired’, and ‘even if you get busy’ [5, 40]. All items except two referred to physical activity (or related words), while two self-efficacy items were phrased neutrally in terms of the outcome behavior [36, 37, 44]. Eight of the 13 self-efficacy items additionally included a physical activity time reference (assessed in minutes), ranging from 10 minutes to 30 minutes (e.g., In the next two hours, I can move for at least 10 minutes’; [12, 38, 39], with some items (*n* = 2 ) also specifying the intensity of physical activity engagement (e.g., ‘I believe I can accumulate at least 30 min of moderate aerobic activity’; [48]). Lastly, one item referred to confidence in enacting one’s plan [36, 37] while another item inquired about confidence in achieving one’s goal [44].

Regarding the response format, most studies used various scale labels/anchors ranging (from, e.g., “not at all true – completely true” [45, 47] or “not at all confident – completely confident” [38, 48]. Further, most studies (*n* = 8/13) used Likert point-scales ranging from 0 up to 10. Two studies applied a visual-analog scale [36–39]. Regarding the sources of the items, it was found that six studies indicated that their items were either adapted or modified from previous research [5, 38–40, 42, 43, 45, 47], followed by three studies that did not indicate where their items derived from [12, 44, 48]. One study specifically designed their items for the respective study [36, 37]. Only three studies reported psychometric properties, all of which applied two items to assess self-efficacy [5, 40, 48]. Lastly, all but one study [44] assessed additional social-cognitive constructs, with intention (*n* = 8; [5, 12, 36–40, 43, 45, 47, 48]) being the most frequently assessed constructs, followed by outcome expectancies (*n* = 1; [5, 40]) and planning (*n* = 3; [36–39, 47]).

#### Statistical Reporting of Within-person Variance Reports

Overall, five studies reported multilevel variance components in the form of intraclass correlation coefficients (ICCs, the amount of between-person variance in relation to total variance) for self-efficacy measures [36, 43, 45, 48], with the remaining studies reporting either beta-coefficients [5, 38–40, 42, 47], odds ratios [44] or mean area under the curve scores ( [37], Table 2). Additionally, one study reported ICCs for physical activity [42] and one for general self-efficacy (i.e., not physical activity related; [38]). ICCs for self-efficacy ranged from approximately 10 to 50% between-person variance. One study reported both between-days (within-subject) variance (6.2%) and within-subject and within-days variance [12], while another calculated ICCs for men and women, separately [45].

**Table 2:** Intraclass correlation coefficients (ICCs) for self-efficacy measures

Study	ICCs for self-efficacy		Assessments per day
	End of day dairy	EMA	
Conroy et al. 2013 [48]	0.49		1
Haag et al. 2023 [36]		0.11	4
Maes et al. 2022 [12]		0.41	6
Maher et al. 2020 [43]		.34^1^	8
Schwaninger et al. 2021 [45]	.39^2^/.48^3^		1

*ICC* Intraclass correlation coefficient; the intraclass correlation stands for the amount of between-person variance in relation to total variance; ^1^only the approach self-efficacy item was considered; ^2^reported for females; ^3^reported for males; *EMA* ecological momentary assessment.

## Discussion

### Summary of Evidence

In this scoping review, we identified 13 primary studies and 13 items assessing momentary self-efficacy published between 2009 and 2025. More than half of the items focused on the near future (i.e., the next few hours, today) and thus applied multiple assessments of self-efficacy per day (four to ten times per day) with fixed intervals between assessments. Only five of the reviewed studies reported ICCs, indicating within- versus between-person variance in self-efficacy, with ICCs varying greatly across studies. Intriguingly, the reporting of ICCs provides the first evidence for self-efficacy to vary within persons across time in their everyday life. While all self-efficacy items semantics focused on participants’ perceived confidence to engage in PA, the items differed in the usage of conditional sentences, the reporting of the origin and psychometrics, the targeted outcome behavior as well as in the intensity and duration of the outcome behavior.

### Assessment Time Span

Most of the reviewed studies assessed self-efficacy dynamically, as indicated by repeated measures every few hours. This seems reasonable, given that more assessments per day are required to adequately capture dynamic within-person processes such as self-efficacy related to physical activity [49]. On average, self-efficacy was assessed six times per day, which is in line with previous reviews on EMA studies in physical activity and diet research in youth revealing seven assessments per day [50] and on physical activity and sedentary behavior that identified five assessments per day [51]. Overall, the more assessments were scheduled per day, the shorter the overall assessment period tended to be. Studies assessing self-efficacy items multiple times per day covered the next two/few hours after the EMA assessment and thus the near future, while studies applying end-of-day/week assessments covered the prospective time span (e.g., tomorrow). Three studies [45, 47, 48] applied end-of-day diaries, in which participants rated their confidence to engage in physical activity for that day.

In addition, most studies reviewed here applied time-based sampling schemes, which is in line with previous research [51]. Time-based approaches generally tend to obtain representative patterns and features of physical activity (and sedentary behavior) over time. Event-based methods in contrast are frequently applied to investigate correlates of physical activity and sedentary behavior [51]. Still, the most impact and insightful study designs combine different sampling strategies (i.e., time-based, random, event-based) to obtain a complete, dynamic picture of how processes unfold in daily life [52].

### Operationalization

In terms of operationalization, the identified items were compared to Bandura’s guidelines [53] for developing self-efficacy items. First, all items were operationalized according to theory, since they judge the participants’ confidence that they can or could do the target behavior [45–47], as opposed to will do, reflecting intentions [53].

Second, most studies used single items to assess self-efficacy, which stands in contrast to Bandura’s guidelines, recommending that self-efficacy measures require a good understanding of the behavioral domain (here physical activity) to accurately measure the multifaceted ways in which self-efficacy beliefs operate (e.g., planning, organization of family obligations).

Third, half of the items included levels of task demands, describing increments of challenges and barriers to successful behavioral engagement (e.g., exercising despite being busy or tired [50]). The obstacles over which self-efficacy beliefs are judged can vary considerably, ranging from psychological (e.g., being worried, stressed), to social (e.g., having friends over, family obligations) and/or contextual conditions (e.g., bad weather, work obligations). As with many behaviors, physical activity engagement is predominantly affected by self-regulation that steers behavioral enactment. Here it is not about whether one can engage in physical activity at times, but more so whether one has the efficacy to do it regularly despite barriers (e.g., being tired). Six of the items here fall into this category of self-efficacy, called self-regulatory efficacy, assessing the broader concept of motivation. In contrast, we also detected items that fall into the category of task self-efficacy, describing beliefs that one can or cannot execute a one-time predefined behavior at different levels of performance (‘I believe I can accumulate at least 30 min of moderate activity tomorrow’ [40]). The main difference is that self-regulatory efficacy reflects a broader skill to deal with various barriers to achieving long-term goals (i.e., sustained physical activity behavior), whereas task self-efficacy mirrors a more focused and specific belief towards a particular behavior (e.g., 30 min of moderate physical activity). The items detected here thus vary on the conceptual level (self-regulatory or task-efficacy) and thus the outcome behavior they assess (repeat- or one-time behavior) and should therefore not be viewed as measurement inconsistencies. In this regard, the same holds true for the items’ assessment windows (i.e., momentary, daily, weekly), which most naturally would result in slight differences in item phrasing.

Fourth, most studies used unipolar response scales with few steps (e.g., 1–5 or 6-point scales), which contrasts with Bandura’s guidelines, recommending unipolar scales that range from 0 “cannot do” to 50 “moderately certain can do” to 100 “highly certain can do” (or 0–10-point scales). As participants tend to avoid extreme endpoints on scales, it remains questionable whether the measures here have been sensitive enough to detect momentary changes in self-efficacy. Fifth, most items concentrated on participants’ perceived capability as of now (i.e., not the future), which aligns with Bandura’s recommendations [53].

Lastly, all included studies reported the specific item(s) used and three quarters reported the source of their items. However, relatively little information was provided regarding the EMA items, where they had been derived from, whether psychometric evaluations had been undertaken, whether they had been modified to suit an EMA design or if content validity was tested. Information regarding the source of the items is particularly interesting, given that relatively few self-reported measures have been validated across EMA studies and populations and items are frequently selected from traditional cross-sectional instruments and adjusted to match the examined timeframe (“in the next 30 minutes”; [51]).

In sum, to improve EMA self-efficacy items, we recommend being mindful of the conceptual level of self-efficacy items, undertaking rigorous conceptual analyses to grasp the multifaceted ways in which efficacy beliefs act (and thus to include more than one item), and ensuring that response formats are unipolar and sensitive enough to detect changes (e.g., 0–100).

### Within-versus Between-person Variance in Self-efficacy in Everyday Life

Overall, half of the studies (5/10 studies) provided multilevel variance components estimated as ICCs for self-efficacy measures and thus indications of how much self-efficacy varied within individuals across situations or time in comparison to between individuals [54]. Translated into practice, the ICCs indicate the degree of the total variance in self-efficacy that is attributable to changes in self-efficacy within the typical participant of a study compared to the degree of the total variance in self-efficacy that is attributable to differences in average self-efficacy between participants in this given study. Put simply, it gives an indication of how much self-efficacy fluctuates within humans within a given timeframe compared to the variance in differences between individuals. Since EMA-based data sets can be analyzed in different ways using either two levels (i.e., moments within individuals) or three-levels (i.e., moments within days within people), within-person variance can be attributed to two sources of variance: within-person across days and variation within-person across moments within days. Just one study identified here [12] reported between-days (within-person) and within-person and within-day variance, showing that the former was lower (6.2%) compared to the latter (52.5%). The other half of the studies reported associations (as opposed to fluctuations) in the form of beta-coefficients. Although beta-coefficients conjecture fluctuations, they do not tell us anything about fluctuations within persons or within a day. Moreover, beta-coefficients are not standardized, which impedes comparisons between studies.

Of those studies that reported ICCs, we identified three EMA-based and two end-of-day-based studies (Table 2). Although we cannot draw any statistical inferences given the low number of studies reporting ICCs, we found a tendency of more within-person variance for momentary compared to daily level assessments of self-efficacy across the studies reviewed. That is, the few existing intensive longitudinal studies suggest that self-efficacy indeed varies considerably within persons across time in everyday life.

Furthermore, while it would be valuable to investigate whether different types of self-efficacy (i.e., task vs. self-regulatory efficacy) fluctuate to varying degrees, our observations suggest that task-efficacy tends to fluctuate more momentarily and within individuals than self-regulatory efficacy (see Table 2). Indeed, task self-efficacy is specific to the demands of a particular task and is therefore affected by factors such as task characteristics (e.g., task difficulty), the environment and its distractions (e.g., presence of others) as well as specific skills required [55]. In contrast, general self-efficacy (i.e., general belief in one’s personal capabilities) is considered relatively stable over time and less sensitive to any specific demands of a given challenge [56]. Further, self-regulatory efficacy may also fluctuate, as it is concerned with self-regulation and emotions – processes that vary and can be dynamically reduced and restored throughout the day by factors like stress, fatigue, and changing contexts [33]. Accordingly, our observations may contribute to theoretical advancements regarding temporal dynamics and timing of effects of self-efficacy by considering the time of day as well as specific task-specific characteristics at different day times as proxies for underlying situational and personal circumstances that could explain variations, depletion, or restoration of self-efficacy [29].

Still, given that none of the studies reporting ICCs included self-regulatory efficacy items measured multiple times per day, and the limited number of studies included, no firm conclusion can be drawn. To extend this discussion, we encourage future EMA-based research investigating within- and between-person differences to report multilevel variance components such as ICCs. Further, we also suggest incorporating the reporting of ICCs into the CREMAs checklist, an adapted STROBE checklist for reporting EMA studies [50].

Lastly, the studies differed greatly in terms of items and study length but only slightly in their sampling schemes (i.e., studies assessed self-efficacy four, six and eight-times per day) which supports the finding of within-person variance across different operationalizations of self-efficacy. However, it remains unclear to what degree fluctuations in self-efficacy were confounded by insufficient reliability. Given that most studies used only one item to assess self-efficacy, reliability indices were not reported, nor did we find information about content analyses. Overall, these findings highlight that intensive longitudinal studies still lack the psychometric foundation underlying most of the traditional “trait” measures of self-efficacy.

### Strengths and Limitations

Some aspects of this review merit further discussion. First, the search strategy was limited to English literature, and no grey literature (e.g., pre-prints, PhD theses) were searched. Non-English publications and unpublished work were consequently not included. However, including grey literature, for which quality standards are not coherently captured, potentially distorts interpretations as non-peer-reviewed and peer-reviewed publications are mixed [57]. Second, the included studies were heterogeneous in terms of sample (i.e., size, sex, age range) and study characteristics (i.e., EMA-based versus end-of-day diaries, number of items used, source of items, psychometrics reported, etc.), resulting in susceptibility to variability across publications. However, given this diversity, our findings should be generalizable to the broad general population.

### Avenues for Future Research and Implications

Given the discrepancy between the high value attributed to micro-temporal within-person processes in current dual-process models on the one hand, and the very few studies reporting self-efficacy fluctuations within-persons on the other hand, future research is warranted to further investigate how self-efficacy unfolds within persons across time. Towards this end and first, future EMA studies would benefit from clearly specifying and reporting study design decisions ranging from the sampling scheme (e.g., multiple assessments versus end-of-day diaries), operationalization (e.g., number of items, validity, origin of items) and statistical details. Second, future study designs may benefit from combining different sampling strategies (i.e., time-based, random, event-based) to obtain a complete dynamic picture of how within-person processes unfold in daily life. In particular, the measurement burst design [58] presents a design type that assesses phenomena repeatedly on macro- and micro-time scales (e.g., daily assessments for 4 weeks (micro-time scale) that are repeated every half a year (macro-time scale)) to analyze intraindividual changes over longer time periods while accounting for intraindividual instability over shorter periods of time. Time scale-separate models that reflect nested effects and interrelations operating at various time scales present another promising possibility. Taking physical activity as an example, on the minute time scale could be bodily movement, which is nested within the bouts of moderate to vigorous physical activity (MVPA) on the hour time scale, which is nested within the minutes of MVPA per day on the daily time scale, which again is nested within minutes of MVPA per week on the weekly time scale etc. (see [28]).

The insights gathered from future research promise to pave the way towards just-in-time-adaptive interventions (JITAI [25]). JITAIs intend to provide the right amount and type of support at the most appropriate time and adjust the intervention content to fluctuations in psychological and contextual states (e.g., providing support when self-efficacy drops to avoid inactivity). This presents an exciting avenue for future work and theory expansion, as recent JITAIs are predominantly steered through behavioral parameters (i.e., periods of prolonged sitting trigger an intervention to engage in physical activity) and the addition of psycho-social parameters, including self-efficacy, promises to be a huge step towards fostering expedient physical activity engagement.

Lastly, the issues identified for self-efficacy measures here (e.g., modification of items, validity), have been shown to be also present for other social-cognitive determinants of physical activity behavior (see [33]). The methods applied in the present review could therefore serve as a template for investigating other determinants of physical activity behavior.

## Conclusion

Although self-efficacy takes a pivotal role as a motivation-oriented construct across various health behavior theories, and micro-temporal within-person processes are at the edge of recent model developments, only few studies have empirically researched how self-efficacy unfolds within-persons across time. This scoping review provides initial evidence that self-efficacy for physical activity varies within-persons across time in everyday life. Moreover, the findings indicate higher within-person variance of self-efficacy on the within- versus between-day level. Since items were assessed dynamically and according to self-efficacy theory (though entailed conceptual differences across studies), the findings appear robust across different self-efficacy operationalizations. However, psychometric features have thus far not been considered in studies, limiting our conclusions. In sum, this scoping review compiles first empirical evidence supporting the core idea of cutting-edge dual process models to focus on micro-temporal within-person processes. Future research is critically warranted to further investigate how self-efficacy unfolds across time, e.g., by applying various sampling strategies and advanced experience sampling designs to inform adaptive and expedient interventions to foster physical activity participation.

## Supplementary Information

Below is the link to the electronic supplementary material.

Supplementary material 1.

## Data Availability

All data used to inform this scoping review are fully disclosed in the manuscript or in its Electronic Supplementary Section. Any other data requirement can be directed to the corresponding author upon reasonable request.

## References

[1] Hajat C, Stein E. The global burden of multiple chronic conditions: a narrative review. Prev Med Rep. 2018;12:284–93. 10.1016/j.pmedr.2018.10.008. 30406006 10.1016/j.pmedr.2018.10.008 PMC6214883

[2] Gutiérrez-Rojas L, Porras-Segovia A, Dunne H, Andrade-González N, Cervilla JA. Prevalence and correlates of major depressive disorder: a systematic review. Braz J Psychiatry. 2020;42:657–72. 10.1590/1516-4446-2020-0650. 32756809 10.1590/1516-4446-2019-0650 PMC7678895

[3] Anderson E, Durstine JL. Physical activity, exercise, and chronic diseases: a brief review. Sports Med Health Sci. 2019;1:3–10. 10.1016/j.smhs.2019.08.006. 35782456 10.1016/j.smhs.2019.08.006 PMC9219321

[4] Kohl HW, Craig CL, Lambert EV, Inoue S, Alkandari JR, Leetongin G, Kahlmeier S. The pandemic of physical inactivity: global action for public health. Lancet. 2012;380:294–305. 10.1016/S0140-6736(12)60898-8. 22818941 10.1016/S0140-6736(12)60898-8

[5] Maher JP, Dzubur E, Huh J, Intille S, Dunton GF. Within-day time-varying associations between behavioral cognitions and physical activity in adults. J Sport Exerc Psychol. 2016;38:423–34. 10.1123/jsep.2016-0058. 27634288 10.1123/jsep.2016-0058 PMC9015818

[6] Bandura A. Health promotion from the perspective of social cognitive theory. In: Abraham C, Conner M, Norman P, editors. Understanding and changing health behaviour: from health beliefs to self-regulation. Amsterdam: Harwood Academic; 2000. pp. 299–339.

[7] Bauman AE, Reis RS, Sallis JF, Wells JC, Loos RJF, Martin BW. Correlates of physical activity: why are some people physically active and others not? Lancet. 2012;380:258–71. 10.1016/S0140-6736(12)60735-1. 22818938 10.1016/S0140-6736(12)60735-1

[8] Nguyen Ho PT, Ha PBT, Tong T, Bramer WM, Hofman A, Lubans DR, et al. Mechanisms linking physical activity with psychiatric symptoms across the lifespan: a systematic review. Sports Med. 2023;53:2171–90. 10.1007/s40279-023-01895-0. 37597100 10.1007/s40279-023-01895-0 PMC10587276

[9] Bandura A. Social cognitive theory: an agentic perspective. Annu Rev Psychol. 2001;52:1–26. 10.1146/annurev.psych.52.1.1. 11148297 10.1146/annurev.psych.52.1.1

[10] Ajzen I. The theory of planned behavior. Organ Behav Hum Decis Process. 1991;50:179–211. 10.1016/0749-5978(91)90020-t.

[11] Rhodes RE, Smith NEI. Personality correlates of physical activity: a review and meta-analysis. Br J Sports Med. 2006;40:958–65. 10.1136/bjsm.2006.028860. 17124108 10.1136/bjsm.2006.028860 PMC2577457

[12] Maes I, Mertens L, Poppe L, Crombez G, Vetrovsky T, van Dyck D. The variability of emotions, physical complaints, intention, and self-efficacy: an ecological momentary assessment study in older adults. PeerJ. 2022;10:e13234. 10.7717/peerj.13234. 35611175 10.7717/peerj.13234 PMC9124457

[13] Young MD, Plotnikoff RC, Collins CE, Callister R, Morgan PJ. Social cognitive theory and physical activity: a systematic review and meta-analysis. Obes Rev. 2014;15:983–95. 10.1111/obr.12225. 25428600 10.1111/obr.12225

[14] Trost SG, Owen N, Bauman AE, Sallis JF, Brown W. Correlates of adults’ participation in physical activity: review and update. Med Sci Sports Exerc. 2002;34:1996–2001. 10.1097/00005768-200212000-00020. 12471307 10.1097/00005768-200212000-00020

[15] Ghayour Baghbani SM, Arabshahi M, Saatchian V. The impact of exercise interventions on perceived self-efficacy and other psychological outcomes in adults: a systematic review and meta-analysis. Eur J Integr Med. 2023;62:102281. 10.1016/j.eujim.2023.102281.

[16] Perski O, Keller J, Kale D, Asare BY-A, Schneider V, Powell D, et al. Understanding health behaviours in context: A systematic review and meta-analysis of ecological momentary assessment studies of five key health behaviours. Health Psychol Rev. 2022;16:576–601. 10.1080/17437199.2022.2112258. 35975950 10.1080/17437199.2022.2112258 PMC9704370

[17] Stults-Kolehmainen MA, Blacutt M, Bartholomew JB, Gilson TA, Ash GI, McKee PC, Sinha R. Motivation States for physical activity and sedentary behavior: desire, urge, wanting, and craving. Front Psychol. 2020;11:568390. 10.3389/fpsyg.2020.568390. 33240154 10.3389/fpsyg.2020.568390 PMC7677192

[18] Ekkekakis P. The Dual-Mode theory of affective responses to exercise in metatheoretical context: I. Initial impetus, basic postulates, and philosophical framework. Int Rev Sport Exerc Psychol. 2009;2:73–94. 10.1080/17509840802705920.

[19] Brand R, Ekkekakis P. Affective–reflective theory of physical inactivity and exercise. Ger J Exerc Sport Res. 2018;48:48–58. 10.1007/s12662-017-0477-9.

[20] Deutsch R, Gawronski B, Hofmann W, editors. Reflective and impulsive determinants of human behavior. New York, London: Routledge; 2017.

[21] Hagger MS. Health behavior and the reflective-impulsive model. In: Deutsch R, Gawronski B, Hofmann W, editors. Reflective and impulsive determinants of human behavior. New York, London: Routledge; 2017. pp. 157–72.

[22] Hagger MS. Non-conscious processes and dual-process theories in health psychology. Health Psychol Rev. 2016;10:375–80. 10.1080/17437199.2016.1244647. 27718880 10.1080/17437199.2016.1244647

[23] Dunton GF. Sustaining health-protective behaviors such as physical activity and healthy eating. JAMA. 2018;320:639–40. 10.1001/jama.2018.6621. 29852046 10.1001/jama.2018.6621 PMC7524543

[24] Nigg CR, Chard C, Zhang G, Nigg C. Children’s physical activity and sedentary behavior is related between different parts of a day. CISS. 2022. 10.36950/2022ciss002.

[25] Nahum-Shani I, Smith SN, Spring BJ, Collins LM, Witkiewitz K, Tewari A, Murphy SA. Just-in-time adaptive interventions (JITAIs) in mobile health: key components and design principles for ongoing health behavior support. Ann Behav Med. 2018;52:446–62. 10.1007/s12160-016-9830-8. 27663578 10.1007/s12160-016-9830-8 PMC5364076

[26] Ebner-Priemer UW, Trull TJ. Ambulatory assessment. Eur Psychol. 2009;14:109–19. 10.1027/1016-9040.14.2.109.

[27] Reichert M, Giurgiu M, Koch E, Wieland LM, Lautenbach S, Neubauer AB, et al. Ambulatory assessment for physical activity research: state of the science, best practices and future directions. Psychol Sport Exerc. 2020. 10.1016/j.psychsport.2020.101742. 32831643 10.1016/j.psychsport.2020.101742 PMC7430559

[28] Spruijt-Metz D, Hekler E, Saranummi N, Intille S, Korhonen I, Nilsen W, et al. Building new computational models to support health behavior change and maintenance: new opportunities in behavioral research. Transl Behav Med. 2015;5:335–46. 10.1007/s13142-015-0324-1. 26327939 10.1007/s13142-015-0324-1 PMC4537459

[29] Scholz U. It’s time to think about time in health psychology. Appl Psychol Health Well Being. 2019;11:173–86. 10.1111/aphw.12156. 30972951 10.1111/aphw.12156

[30] Timm I, Giurgiu M, Ebner-Priemer U, Reichert M. The within-subject association of physical behavior and affective well-being in everyday life: a systematic literature review. Sports Med. 2024;54:1667–705. 10.1007/s40279-024-02016-1. 38705972 10.1007/s40279-024-02016-1 PMC11239742

[31] Dunton GF, Liao Y, Kawabata K, Intille S. Momentary assessment of adults’ physical activity and sedentary behavior: feasibility and validity. Front Psychol. 2012;3:260. 10.3389/fpsyg.2012.00260. 22866046 10.3389/fpsyg.2012.00260 PMC3408114

[32] Chevance G, Baretta D, Heino M, Perski O, Olthof M, Klasnja P, et al. Characterizing and predicting person-specific, day-to-day, fluctuations in walking behavior. PLoS ONE. 2021;16:e0251659. 10.1371/journal.pone.0251659. 33989338 10.1371/journal.pone.0251659 PMC8121346

[33] Bittel KM, O’Briant KY, Ragaglia RM, Buseth L, Murtha C, Yu J, et al. Associations between social cognitive determinants and movement-related behaviors in studies using ecological momentary assessment methods: syst rev. JMIR MHEALTH UHEALTH. 2023;11:e44104. 10.2196/44104. 37027185 10.2196/44104 PMC10131703

[34] Tricco AC, Lillie E, Zarin W, O’Brien KK, Colquhoun H, Levac D, et al. PRISMA extension for scoping reviews (PRISMA-ScR): checklist and explanation. Ann Intern Med. 2018;169:467–73. 10.7326/M18-0850. 30178033 10.7326/M18-0850

[35] Lim WM, Weissmann MA. Toward a theory of behavioral control. J Strateg Mark. 2023;31:185–211. 10.1080/0965254X.2021.1890190.

[36] Haag D, Carrozzo E, Pannicke B, Niebauer J, Blechert J. Within-person association of volitional factors and physical activity: insights from an ecological momentary assessment study. Psychol Sport Exerc. 2023;68:102445. 10.1016/j.psychsport.2023.102445. 37665897 10.1016/j.psychsport.2023.102445

[37] Kumar D, Haag D, Blechert J, Niebauer J, Smeddinck JD. Feature selection for physical activity prediction using ecological momentary assessments to personalize intervention timing: longitudinal observational study. JMIR MHEALTH UHEALTH. 2025;13:e57255. 10.2196/57255. 39865572 10.2196/57255 PMC11785349

[38] Maher JP, Labban JD, Hudgins BL, Hevel DJ, Bittel KM, Kennedy-Malone L, Hedeker D. Moving beyond mean levels: associations between subject-level variability in psychological determinants and physical activity in older adults. J Phys Act Health. 2025;22:112–22. 10.1123/jpah.2024-0350. 39504953 10.1123/jpah.2024-0350

[39] Maher JP, Behler MH, Hevel DJ, Hudgins BL, Kennedy-Malone L, Khan IF, et al. Determinants of physical activity adoption and maintenance in older adults: a dual process approach. Psychol Sport Exerc. 2025;77:102800. 10.1016/j.psychsport.2024.102800. 39722313 10.1016/j.psychsport.2024.102800 PMC11781950

[40] Pickering TA, Huh J, Intille S, Liao Y, Pentz MA, Dunton GF. Physical activity and variation in momentary behavioral cognitions: an ecological momentary assessment study. J Phys Act Health. 2016;13:344–51. 10.1123/jpah.2014-0547. 26284314 10.1123/jpah.2014-0547

[41] Geeraerts D, editor. Oxford research encyclopedia of linguistics. Oxford University Press; 2017.

[42] Dunton GF, Atienza AA, Castro CM, King AC. Using ecological momentary assessment to examine antecedents and correlates of physical activity bouts in adults age 50 + years: a pilot study. Ann Behav Med. 2009;38:249–55. 10.1007/s12160-009-9141-4. 20052568 10.1007/s12160-009-9141-4 PMC7155921

[43] Maher JP, Dunton GF. Within-day time-varying associations between motivation and movement-related behaviors in older adults. Psychol Sport Exerc. 2020;47:101522. 10.1016/j.psychsport.2019.04.012.

[44] Oh YJ, Hoffmann TJ, Fukuoka Y. A novel approach to assess weekly self-efficacy for meeting personalized physical activity goals via a cellphone: 12-week longitudinal study. JMIR Form Res. 2023;7:e38877. 10.2196/38877. 36705945 10.2196/38877 PMC9919464

[45] Schwaninger P, Berli C, Lüscher J, Scholz U. Cultivation or enabling? Day-to-day associations between self-efficacy and received support in couples. Soc Sci Med. 2021;287:114330. 10.1016/j.socscimed.2021.114330. 34455336 10.1016/j.socscimed.2021.114330

[46] Schwarzer R. Modeling health behavior change: how to predict and modify the adoption and maintenance of health behaviors. Appl Psychol. 2008;57:1–29. 10.1111/j.1464-0597.2007.00325.x.

[47] Berli C, Lüscher J, Luszczynska A, Schwarzer R, Scholz U. Couples’ daily self-regulation: the health action process approach at the dyadic level. PLoS ONE. 2018;13:e0205887. 10.1371/journal.pone.0205887. 30372470 10.1371/journal.pone.0205887 PMC6205589

[48] Conroy DE, Elavsky S, Doerksen SE, Maher JP. A daily process analysis of intentions and physical activity in college students. J Sport Exerc Psychol. 2013;35:493–502. 10.1123/jsep.35.5.493. 24197717 10.1123/jsep.35.5.493 PMC4104787

[49] Conroy DE, Elavsky S, Hyde AL, Doerksen SE. The dynamic nature of physical activity intentions: a within-person perspective on intention-behavior coupling. J Sport Exerc Psychol. 2011;33:807–27. 10.1123/jsep.33.6.807. 22262706 10.1123/jsep.33.6.807 PMC4137572

[50] Liao Y, Skelton K, Dunton G, Bruening M. A systematic review of methods and procedures used in ecological momentary assessments of diet and physical activity research in youth: an adapted STROBE checklist for reporting EMA studies (CREMAS). J Med Internet Res. 2016;18:e151. 10.2196/jmir.4954. 27328833 10.2196/jmir.4954 PMC4933800

[51] Degroote L, DeSmet A, de Bourdeaudhuij I, van Dyck D, Crombez G. Content validity and methodological considerations in ecological momentary assessment studies on physical activity and sedentary behaviour: a systematic review. Int J Behav Nutr Phys Act. 2020;17:35. 10.1186/s12966-020-00932-9. 32151251 10.1186/s12966-020-00932-9 PMC7063739

[52] Trull TJ, Ebner-Priemer UW. Ambulatory assessment in psychopathology research: a review of recommended reporting guidelines and current practices. J Abnorm Psychol. 2020;129:56–63. 10.1037/abn0000473. 31868388 10.1037/abn0000473

[53] Bandura A. Guide for constructing self-efficacy scales. In: Urdan T, Pajares F, editors. Self-Efficacy Beliefs of Adolescents: IAP; 2006. pp. 307–337.

[54] Scott SB, Sliwinski MJ, Zawadzki M, Stawski RS, Kim J, Marcusson-Clavertz D, et al. A coordinated analysis of variance in affect in daily life. Assessment. 2020;27:1683–98. 10.1177/1073191118799460. 30198310 10.1177/1073191118799460 PMC6408986

[55] Judge TA, Jackson CL, Shaw JC, Scott BA, Rich BL. Self-efficacy and work-related performance: the integral role of individual differences. J Appl Psychol. 2007;92:107–27. 10.1037/0021-9010.92.1.107. 17227155 10.1037/0021-9010.92.1.107

[56] Schwarzer R, Luszczynska A. Self-efficacy. In: Ruch W, Bakker AB, Tay L, Gander F, editors. Handbook of positive psychology assessment. Hogrefe Publishing GmbH; 2022. pp. 207–17.

[57] Mahood Q, van Eerd D, Irvin E. Searching for grey literature for systematic reviews: challenges and benefits. Res Synth Methods. 2014;5:221–34. 10.1002/jrsm.1106. 26052848 10.1002/jrsm.1106

[58] Nesselroade JR. The warp and the woof of the developmental fabric. In: Liben LS, Palermo DS, editors. Visions of Aesthetics, the environment & development: the legacy of Joachim F. Wholwill. Hoboken: Taylor and Francis; 2013. pp. 213–40.

[59] Atienza AA, Collins R, King AC. The mediating effects of situational control on social support and mood following a stressor: a prospective study of dementia caregivers in their natural environments. J Gerontol B Psychol Sci Soc Sci. 2001;56:129-39. 10.1093/geronb/56.3.s129 10.1093/geronb/56.3.s129 11316838

[60] Scholz U, Nagy G, Gööhner W, Luszczynska A, Kliegel M. Changes in self-regulatory cognitions as predictors of changes in smoking and nutrition behaviour. Psychol Health. 2009;24:545-61. 10.1080/08870440801902519 10.1080/08870440801902519 20205011

