# Supplementary material for: Daily Life Studies on Dynamic Within-person Fluctuations of Self-efficacy in the Physical Activity Context: A Scoping Review
Source: Sports Med Open. 2026 Feb 7;12:9. doi: 10.1186/s40798-025-00973-z (PMC12882901; doi:10.1186/s40798-025-00973-z)
Supplement: Supplementary file 1 — Supplementary material 1. [file 40798_2025_973_MOESM1_ESM.docx]

**Title:** Daily life studies on dynamic within-person fluctuations of self-efficacy in the physical activity context: a scoping review

Journal name: Sports Medicine - Open

**Electronic supplementary material**

**ESM 1. Methods**

**ESM Sect. 1.1**

**Table 1. Inclusion criteria and guidance screening chart.**

| **Criteria** | **Guidance** |
| --- | --- |
| Ecological Momentary Assessment | Uses an EMA methodology of any length |
| Physical Activity OR Exercise Behavior | Measure can be subjective or objective  Can be a continuous monitoring OR measured with EMA prompts  Must have at least one measurement (or have continuous measurement on each EMA day) |
| Self-Efficacy | Assessed based on social cognitive theory (Bandura, 1977) – i.e., similar concepts (e.g., perceived behavioral control, competence) were excluded  Must be measured at least once per EMA day  Excluded if baseline measure only |
| Participants | 18+ years  Cannot be in a current clinical population (e.g., inpatient patients) |
| Full Paper Availability | Excluded if only conference abstracts, no results available (e.g., feasibility studies) |
| English Language | Excluded if not available in English |

Note. EMA = ecological momentary assessment

**ESM Sect. 1.2 Search strategy**

- Free text terms were searched using Boolean operators applied to the title or abstract
- Three groups of terms were combined: the first group of terms was relevant to ecological momentary assessment and within-person study designs; the second group referred to physical activity and exercise behavior and the last group was relevant to self-efficacy.
- Truncations were applied to include multiple word endings
- Group 1: (“ecological momentary assessment*”) OR (“intensive longitudinal”) OR (“ambulatory assessment*”) OR (“experience sampl*”) OR (“daily diar*”) OR (“ecological momentary intervention”) OR within-person OR within-subject* OR idiographic OR intraindividual OR “real time data capture” OR “daily life research”
- Group 2: “physical activit*” OR exercis*
- Group 3: self-efficacy

The following search strings for each database are provided in supplementary Table 2.

**Supplementary Table 2. Search strings per database**

| **Database** | **Search string** | **Limiters** |
| --- | --- | --- |
| PsycInfo, PsycArticles | (TI (("ecological momentary assessment*") OR AB ("ecological momentary assessment*") OR TI ("intensive longitudinal") OR AB ("intensive longitudinal") OR TI ("ambulatory assessment*") OR AB ("ambulatory assessment*") OR TI ("experience sampl*") OR AB ("experience sampl*") OR TI ("daily diar*") OR AB ("daily diar*") OR TI ("ecological momentary intervention") OR AB ("ecological momentary intervention") OR TI ("within-person") OR AB ("within-person") OR TI ("within-subject*") OR AB ("within-subject*") OR TI ("idiographic") OR AB ("idiographic") OR ("intraindividual") OR AB ("intraindividual") OR TI ("real time data capture") OR AB ("real time data capture") OR TI ("real time data capture") OR AB ("real time data capture")) ) AND ( TI (("physical activit*") OR AB ("physical activit*") OR TI ("exerci*") AB ("exerci*") ) ) AND ( TI ("self-efficacy") OR AB ("self-efficacy") ) | Full Text; Scholarly (Peer Reviewed) Journals; Age Groups: Adulthood (18 yrs & older); Publication Type: Peer Reviewed Journal; English; Language: English; Age Groups: Adulthood (18 yrs & older); Language: English |
| SPortDiscus | (TI (("ecological momentary assessment*") OR AB ("ecological momentary assessment*") OR TI ("intensive longitudinal") OR AB ("intensive longitudinal") OR TI ("ambulatory assessment*") OR AB ("ambulatory assessment*") OR TI ("experience sampl*") OR AB ("experience sampl*") OR TI ("daily diar*") OR AB ("daily diar*") OR TI ("ecological momentary intervention") OR AB ("ecological momentary intervention") OR TI ("within-person") OR AB ("within-person") OR TI ("within-subject*") OR AB ("within-subject*") OR TI ("idiographic") OR AB ("idiographic") OR ("intraindividual") OR AB ("intraindividual") OR TI ("real time data capture") OR AB ("real time data capture") OR TI ("real time data capture") OR AB ("real time data capture")) ) AND ( TI (("physical activit*") OR AB ("physical activit*") OR TI ("exerci*") AB ("exerci*") ) ) AND ( TI ("self-efficacy") OR AB ("self-efficacy") ) | Full Text; Scholarly (Peer Reviewed) Journals; Age Groups: Adulthood (18 yrs & older); Publication Type: Peer Reviewed Journal; English; Language: English; Age Groups: Adulthood (18 yrs & older); Language: English |
| PubMed | (((((( AND (( "physical activit*" OR "exerci*" )) AND ("self-efficacy") AND ((fft[Filter]) AND (english[Filter]) AND (alladult[Filter]))) AND ("ecological momentary assessment*"[Title/Abstract] OR "intensive longitudinal"[Title/Abstract] OR "ambulatory assessment*"[Title/Abstract] OR "experience sampl*"[Title/Abstract] OR "daily diar*"[Title/Abstract] OR "ecological momentary intervention"[Title/Abstract] OR "within-person"[Title/Abstract] OR "within-subject*"[Title/Abstract] OR "idiographic"[Title/Abstract] OR "intraindividual"[Title/Abstract] OR "real time data capture"[Title/Abstract] OR "daily life research"[Title/Abstract]))))) AND ("physical activit*"[Title/Abstract] OR "exerci*"[Title/Abstract])) AND ("self-efficacy"[Title/Abstract]) | Filters: Full text, English, Adult: 19+ years, Full text, English, Adult: 19+ years |

**EMS 2. Results**
**ESM Sect. 2.1**

**Supplementary Table 3 Study and sample characteristics**

| **Author** | **Participants**  **(N, gender, age)** | **Theoretical background** | **Intervention** | **Study length** | **Technology used** | **PA measure** |
| --- | --- | --- | --- | --- | --- | --- |
| Berli et al., 2018 [47] | Inactive, overweight couples, N = 61 couples, 44.23 yr | HAPA | No | 28 days | Web-based questionnaire | Accelerometer (daily MVPA) |
| Conroy et al. 2013 [48] | College students, N = 63,  F = 37, | TPB | No | 14 days | Web-based questionnaire | IPAQ (daily PA) |
| Dunton et al., 2009 [42] | Healthy, community-dwelling middle-aged to older adults, N = 23, F=16, 60.65 yr | Not specified | No | 14 days | Handheld electronic diaries | MVPA via EMA |
| Haag et al., 2023 [36] | Healthy adults, N = 35,  F = 25, 39.14 yr | HAPA | No | 21 days | EMA app | PA (EMA) |
| Kumar et al., 2025 [37] | Healthy adults, N = 43, F = 31,  39.14 yr | HAPA | No | 21 days | EMA app | PA (EMA) |
| Maes et al., 2022 [12] | Healthy older adults, N = 64, F = 36, 72.1 yr | COM-B framework | No | 7 days | Smartphone based |  |
| Maher et al., 2016 [5] | Community-dwelling adults, N = 116, F = 86, 40.3 yr  Project MOBILE | SCT | No | 3 x 4-day measurement bursts with 6 months between bursts | Mobile phone with installed software | Accelerometer (Actigraph) |
| Maher et al., 2020 [43] | Older adults, N = 104, F = 65, 72.4 yr | SCT | No | 10 days | Mobile phone | activePAL accelerometer |
| Maher et al., 2025 [38] | Older adults, N = 202, F = 143, 69.98 yr  Project SMART | Not specified | No | 3 x 4-day measurement bursts with 6 months between bursts | Smartphone based | Actigraph GT3X |
| Maher et al., 2025 [39] | Older adults, N = 202, N = 104, F = 65, 72.4 yr  Project SMART | Dual process models | No | 3 x 4-day measurement bursts with 6 months between bursts | Smartphone based | Actigraph GT3X |
| Oh et al., 2023 [40] | Community-dwelling women, N = 140, 52.7 yr | SCT | Yes, PA intervention | 12 weeks | Study mobile app | Accelerometer |
| Pickering et al., 2016 [40] | Community-dwelling adults, N = 116, F = 84, 40.3 yr  Project MOBILE | SCT | No | 4 days (2 weekdays, 2 weekend days) | Mobile phone with installed software | Accelerometer (Actigraph) |
| Schwaninger et al., 2021 [45] | Overweight heterosexual couples, N = 99, F 45.31 yr, M = 47.29 yr | SCT (the enabling and the cultivating hypothesis) | Yes, secondary analysis of data collected at 6 months follow-up of larger RCT to promote PA | 14 days | Smartphone based | Triaxial GT3X + monitors (ActiGraph) |

Notes: COM-B = capability, opportunity, motivation, and behavior framework. EMA = ecological momentary assessment. F = female. HAPA = health action process approach. IPAQ = international physical activity questionnaire. MVPA = moderate to vigorous physical activity. PA = physical activity. SCT = social-cognitive theory. TPB = theory of planned behavior. yr = years
